# The Teacher, the Physician and the Person: How Faculty's Teaching Performance Influences Their Role Modelling

**DOI:** 10.1371/journal.pone.0032089

**Published:** 2012-03-12

**Authors:** Benjamin C. M. Boerebach, Kiki M. J. M. H. Lombarts, Christiaan Keijzer, Maas Jan Heineman, Onyebuchi A. Arah

**Affiliations:** 1 Department of Quality Management and Process Innovation, Academic Medical Center, University of Amsterdam, Amsterdam, The Netherlands; 2 Department of Anesthesiology, Academic Medical Center, University of Amsterdam, Amsterdam, The Netherlands; 3 Department of Obstetrics and Gynecology, Academic Medical Center, University of Amsterdam, Amsterdam, The Netherlands; 4 Department of Epidemiology, School of Public Health, University of California Los Angeles, Los Angeles, California, United States of America; 5 UCLA Center for Health Policy Research, Los Angeles, California, United States of America; Sapienza University of Rome, Italy

## Abstract

**Objective:**

Previous studies identified different typologies of role models (as teacher/supervisor, physician and person) and explored which of faculty's characteristics could distinguish good role models. The aim of this study was to explore how and to which extent clinical faculty's teaching performance influences residents' evaluations of faculty's different role modelling statuses, especially across different specialties.

**Methods:**

In a prospective multicenter multispecialty study of faculty's teaching performance, we used web-based questionnaires to gather empirical data from residents. The main outcome measures were the different typologies of role modelling. The predictors were faculty's overall teaching performance and faculty's teaching performance on specific domains of teaching. The data were analyzed using multilevel regression equations.

**Results:**

In total 219 (69% response rate) residents filled out 2111 questionnaires about 423 (96% response rate) faculty. Faculty's overall teaching performance influenced all role model typologies (OR: from 8.0 to 166.2). For the specific domains of teaching, overall, all three role model typologies were strongly associated with “professional attitude towards residents” (OR: 3.28 for teacher/supervisor, 2.72 for physician and 7.20 for the person role). Further, the teacher/supervisor role was strongly associated with “feedback” and “learning climate” (OR: 3.23 and 2.70). However, the associations of the specific domains of teaching with faculty's role modelling varied widely across specialties.

**Conclusion:**

This study suggests that faculty can substantially enhance their role modelling by improving their teaching performance. The amount of influence that the specific domains of teaching have on role modelling differs across specialties.

## Introduction

An important part of the learning process of residents occurs through observation and imitation of more experienced faculty, who act as role models. The importance of good role modelling in residency training is globally understood and is believed to be an important teaching method in shaping the values, attitudes, behaviour, and ethics of residents [1]–[3]. Role modelling can be seen as an overarching activity that encompasses everything faculty do in their being and acting as professionals [1]. Insight in methods to improve good role modelling could be of great interest to clinical faculty.

**Figure 1:**
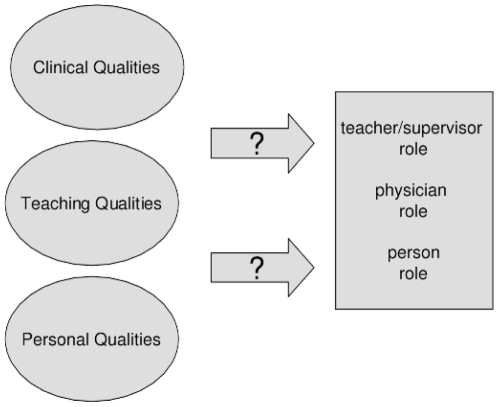
Relationship between role model characteristics and the role model typologies.

Previous studies identified different components of role modelling [4] resulting in various typologies of role models as a *teacher/supervisor, physician* and *person* (see box S1) [5]. Equally, the specific characteristics of role models can be categorized into three different categories: *clinical qualities, teaching qualities* and *personal qualities* [1], [6]. However, the relationship between the distinctive roles and role model's characteristics is largely unknown (figure 1). There seems to be no one-on-one relationship, as some clinical and personal qualities have been shown to influence all distinctive roles simultaneously [7] and teaching qualities have been shown to influence the physician role [8]. These findings raise the question how the distinctive roles are influenced by different role model characteristics.

Although a few empirical studies identified some teaching qualities that could influence role modelling [3], [8], [9], these studies do not distinguish between the various role modeling typologies. Besides they do not study differences across specialties. The aim of this study is to provide clinical faculty with specific insight in how their teaching performance could influence their being seen as different kinds of role models by residents. More specifically, this study wants to explore 1) through which of the distinctive roles teaching performance influence role modelling, 2) if this occurs similarly across specialties. To answer these research questions, we used the Systematic Evaluation of Teaching Qualities (SETQ) system to obtain faculty's teaching performance data [10]–[12].

## Materials and Methods

### Study Population and Setting

To address the objective of this study, we gathered quantitative empirical data. We collected the data using web-based questionnaires filled out by residents. Data were collected between September and November 2010. In total 317 residents of 17 different residency training programs (in Anesthesiology, Internal medicine, Obstetrics & Gynecology, Pediatrics and Surgery) were invited to fill out the questionnaires. The residency programs were situated in three academic (eight programs) and eight non-academic (nine programs) teaching hospitals.

Residency training in The Netherlands is a joint responsibility of several teaching faculty who form an educational team. The educational teams of the residency training programs included in this study ranged from 6 to 87 faculty per team. In some larger educational teams, smaller sub-teams are formed to guarantee the personal interaction between faculty and residents. At the sub-teams of pediatrics, surgery and internal medicine, these sub-teams may also focus on different subspecialties. Because of this setting, residents could choose which and how many faculty to evaluate, based on whose teaching performance the resident felt he or she was able to evaluate accurately. In total, 441 faculty could be evaluated. Participants were invited to participate via email. The invitation email mentioned the formative purpose and use of the evaluations and stressed the confidential and voluntary character of participation.

### Study design and Questionnaires

For measurement of faculty's teaching performance, the *System for Evaluation of Teaching Qualities* (SETQ) questionnaires, which are based on the Stanford Faculty Development Program (SFDP-26) questionnaires [13], were used. SETQ is a dynamic system developed for the continuous evaluation and development of faculty involved in teaching residents and is widely used in The Netherlands [11]. The questionnaires are developed and validated for different specialties and evaluate faculty's teaching performance in five domains of teaching: *learning climate, professional attitude towards residents, communication of goals, evaluation of residents* and *feedback* [10]–[12]. All questionnaires contain 20 “generic items”, for some specialties the questionnaires contain additional items (see appendix S1). To obtain reliable SETQ evaluation data on each faculty (predicted Cronbach's alpha of all individual domains >0.70), at least six resident evaluations are needed for anesthesiology [11], five for internal medicine and pediatrics [10], four for obstetrics and gynecology [12] and seven for surgery (unpublished study).

To evaluate faculty's role modelling, additional questions were formulated after discussing faculty's role modelling with a group of 15 anesthesiology residents. Based on the literature we initially proposed a four role model typology (physicians, person, teacher and supervisor) [5], [9] and discussed these typologies as described in the literature. Because in residents' perception the roles teacher and supervisor could not be adequately distinguished in their daily residency training, the initial four role model typology was than reduced to contain only three role models. A similar classification has been used in previous studies [4], [7], [14].

### Outcome variables

The outcome variables were residents' perception of faculty's role modelling on the three different *role models typologies*. At the end of the SETQ questionnaire each respondent was asked to answer three questions about faculty's role modelling. These items were: *During my residency, this faculty is a role model to me in his/her role as… (Q1: teacher/supervisor, Q2: physician, Q3: a person)*. The items were scored on 5-point Likert scale: 1 = “strongly disagree”, 2 = “disagree”, 3 = “neutral”, 4 = “agree”, 5 = “strongly agree”, and there was an additional option “I can not judge”. Each item was preceded by some examples of typical skills and characteristics for this role model typology (see box S2).

### Main predictors

**Figure 2:**
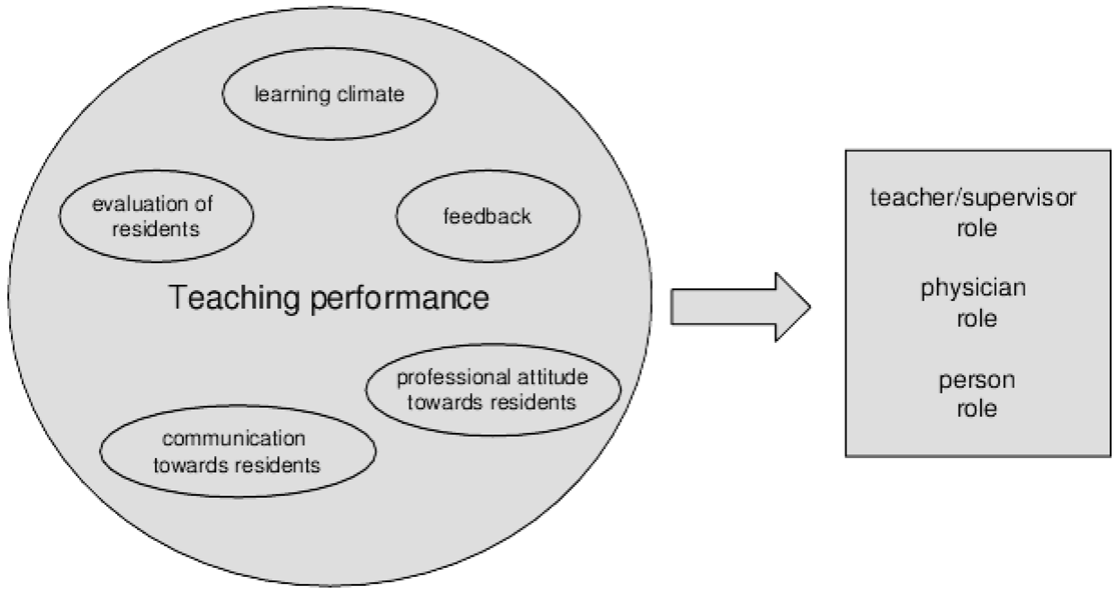
The predictors and outcome variables of the regression equations.

The main predictor was faculty's teaching performance, as evaluated by residents via the SETQ questionnaires. In the analyses, we included faculty's *overall teaching performance*, which is defined as the mean score of all the SETQ items (appendix S1), as a predictor. We also included faculty's teaching performance on the five previously defined SETQ domains as predictors (see figure 2). The items of the SETQ questionnaires were scored on the same 5-point Likert scale as the role model items.

### Covariates

The analytical models used to analyze the associations between the predictors and the outcome variables, were adjusted for several covariates: faculty's sex and experience, resident's sex and residency year (all models) and hospital and specialty (only in the models where more than one department and/or specialty was included) [15]. We performed the analyses both adjusted and unadjusted for these covariates to explore if they confounded the associations between teaching performance and the role model typologies.

### Analytical Strategies

We were aware that cross-clustering could affect the associations between teaching performance and the role modelling typologies, because in both the evaluating (residents) and evaluated (faculty) participants, there was potential clustering. More specifically, if faculty were evaluated by more then one resident in their residency program, the associations could be stronger within that individual faculty than across faculty members. Additionally, associations of scores given by the same resident, could be stronger compared to associations of scores given across residents. Because the data contained both nominal and continuous variables, we used ordinal logistic generalized estimating equation (GEE) models to study associations between faculty's teaching performance and the role model typologies. These regression equations can analyze both nominal and continuous variables simultaneously and allow for appropriate adjustment of cross-clustering.

However, before tackling our main objectives, we used descriptive statistics to describe the participants' characteristics. Besides, we described median and mean scores of residents' ratings of faculty on the three role model items.

To explore the association between faculty's overall teaching performance and the distinctive type of role models we used GEE models with faculty's *overall teaching performance* as predictor and the role modelling items as outcome variables. Similarly, we used GEE models with faculty's performance on *specific domains of teaching* as predictors, to explore if these domains influenced the role model typologies. Estimated odd ratios and their 95% confidence interval were reported. Data were analyzed using statistical package PASW Statistics 18.0.2 for windows (SPSS Inc., 2009).

## Results

In total, 219 (69%) residents filled out 2111 questionnaires about 423 (96%) faculty (see table 1 for participants' characteristics). The residents who participated in this study were equally divided over different phases of their residency training (1^st^ year: 19%, 2^nd^: 14%, 3^rd^: 16%, 4^th^: 24%, 5^th^: 17%, 6^th^: 9%).

**Table 1:** Characteristics of study participants.

	All specialties	Anesthesiology	Internal medicine	Obstetrics & Gynecology	Pediatrics	Surgery
Number of residency programs	17	1	1	9	4	2
Number of faculty evaluated (% of the faculty that could be evaluated)	423 (96%)	42 (100%)	56 (88%)	110 (96%)	178 (97%)	37 (100%)
Number of residents (% of the residents invited)	219 (69%)	25 (68%)	40 (73%)	64 (72%)	69 (65%)	21 (72%)
Number of evaluations	2111	362	263	532	670	284
Median number of evaluations per faculty (min-max)	4 (1–20)	8 (3–19)	3 (1–18)	5 (1–11)	2.5 (1–20)	7 (5–12)
Percentage of female faculty	45.6%	34.1%	40.4%	52.8%	51.9%	16.7%
Percentage of female residents	61.5%	64.0%	52.5%	71.9%	64.7%	33.3%
Experience: Mean number of years in practice since fist registration an specialist (± SD)	12 (±9)	13 (±10)	13 (±10)	13 (±10)	11 (±9)	13 (±10)
Mean faculty age (± SD)	46.7 (±8.5)	49.3 (±8.4)	47.3 (±8.9)	48.3 (±8.5)	44.6 (±8.0)	48.1 (±9.0)

Further, the median and mean scores given by the residents on the different role model typologies indicate that residents rate their teaching faculty higher on the physician role, compared to the teacher/supervisor and person role (table 2).

**Table 2:** Median and mean score of residents' ratings of the items: During my residency, this faculty is a role model to me in his/her role as… [1: teacher/supervisor, 2: physician and 3: person].

	Teacher/Supervisor		Physician		Person	
	Median (20^th^–80^th^ percentile)	Mean	Median (20^th^–80^th^ percentile)	Mean	Median (20^th^–80^th^ percentile)	Mean
All Specialties	4.00 (3.00–5.00)	3.80	4.00 (3.00–5.00)	4.00	4.00 (3.00–5.00)	3.81
Anesthesiology	4.00 (3.00–4.00)	3.56	4.00 (3.00–4.00)	3.74	4.00 (3.00–4.00)	3.58
Internal medicine	4.00 (4.00–5.00)	4.14	4.00 (4.00–5.00)	4.25	4.00 (3.00–5.00)	4.03
Gynecology & Obstetrics	4.00 (3.00–5.00)	3.82	4.00 (3.00–5.00)	4.00	4.00 (3.00–5.00)	3.91
Pediatrics	4.00 (3.00–5.00)	3.86	4.00 (4.00–5.00)	4.09	4.00 (3.00–5.00)	3.91
surgery	4.00 (3.00–4.00)	3.64	4.00 (3.00–5.00)	3.89	4.00 (3.00–4.00)	3.64

The odd ratios for the adjusted associations between *overall teaching performance* and the different type of role models were consistently highest for the role of teacher/supervisor over all specialties (OR: from 47.3 to 166.2), see table 3. The odd ratios for the adjusted associations between the specific domains of teaching and the role modelling items differed per domain, type of role model and specialty (see table 4). Overall, *professional attitude towards residents* and *feedback* had the highest odd ratios for the teacher/supervisor the person role, while *professional attitude towards residents* and *evaluation of residents* had highest odd ratios for the physician role. However, there were considerable differences across specialties. For all analyses, the unadjusted models differed only marginally from the adjusted models, so only the results of the adjusted models are shown (table 3 and 4).

**Table 3:** Odds ratios (OR) for the adjusted associations between faculty's Teaching Performance and different types of role models as seen by the residents.

	Teacher/Supervisor: OR (95% C.I.)	Physician: OR (95% C.I.)	Person: OR (95% C.I.)
All Specialties#	73.6 (54.8–98.8)	15.5 (12.3–19.5)	13.8 (11.2–17.0)
Anesthesiology	47.9 (23.7–96.8)	9.7 (5.8–16.4)	8.0 (5.2–12.4)
Internal medicine	47.3 (21.6–103.6)	10.8 (5.9–19.7)	15.9 (8.8–28.8)
Gynecology & Obstetrics*	75.2 (45.3–124.7)	16.2 (10.5–25.0)	16.6 (10.8–25.5)
Pediatrics*	166.2 (87.9–314.3)	23.9 (14.9–38.2)	16.1 (10.9–23.6)
Surgery*	133.9 (47.3–378.8)	30.3 (13.8–66.5)	56.5 (25.7–124.3)

All models are adjusted for residents' residency training year and sex and for faculty's experience and sex.

* additionally adjusted for hospital.

# additionally adjusted for specialty and hospital.

**Table 4:** Odds ratios (OR) for the adjusted associations between faculty's specific domains of teaching performance and the different types of role models as seen by the residents.

	Teacher/Supervisor: OR (95% C.I.)	Physician: OR (95% C.I.)	Person: OR (95% C.I.)
*All Specialties* #			
Learning climate	2.70 (2.03–3.60)	1.76 (1.33–2.33)	1.38 (1.05–1.83)
Professional attitude towards residents	3.28 (2.55–4.21)	2.72 (2.14–3.45)	7.20 (5.50–9.43)
Communication of goals	1.64 (1.32–2.04)	1.25 (1.02–1.52)	1.27 (1.05–1.54)
Evaluation of residents	1.89 (1.42–2.51)	2.08 (1.59–2.72)	0.93 (0.72–1.20)
Feedback	3.23 (2.47–4.23)	1.40 (1.09–1.80)	2.20 (1.73–2.80)
*Anesthesiology*			
Learning climate	1.55 (0.85–2.85)	0.77 (0.45–1.34)	0.65 (0.38–1.11)
Professional attitude towards residents	2.22 (1.34–3.69)	3.20 (2.02–5.39)	5.55 (3.30–9.35)
Communication of goals	2.09 (1.32–3.29)	1.09 (0.68–1.74)	1.43 (0.95–2.16)
Evaluation of residents	2.04 (1.11–3.75)	3.19 (1.79–5.71)	1.57 (0.93–2.64)
Feedback	3.95 (2.17–7.19)	1.94 (1.09–3.45)	2.52 (1.42–4.49)
*Internal medicine*			
Learning climate	5.97 (2.22–16.10)	1.72 (0.81–3.64)	2.21 (0.90–5.40)
Professional attitude towards residents	3.82 (1.59–9.17)	1.94 (0.98–3.83)	6.92 (3.38–14.19)
Communication of goals	1.29 (0.65–2.57)	1.51 (0.78–2.95)	1.11 (0.55–2.24)
Evaluation of residents	1.03 (0.39–2.69)	1.54 (0.70–3.39)	1.32 (0.60–2.91)
Feedback	2.33 (1.16–4.72)	1.23 (0.70–2.16)	1.38 (0.79–2.39)
*Gynecology & Obstetrics* *			
Learning climate	2.27 (1.34–3.85)	1.99 (1.14–3.46)	1.55 (0.96–2.50)
Professional attitude towards residents	3.58 (2.31–5.56)	3.21 (2.06–5.00)	9.31 (5.63–15.39)
Communication of goals	1.09 (0.74–1.60)	1.17 (0.78–1.74)	1.10 (0.77–1.58)
Evaluation of residents	2.67 (1.57–4.53)	1.95 (1.17–3.26)	0.80 (0.49–1.29)
Feedback	4.40 (2.66–7.29)	1.35 (0.88–2.08)	2.41 (1.61–3.60)
*Pediatrics* *			
Learning climate	6.50 (2.80–13.09)	3.67 (1.87–7.24)	1.82 (0.98–3.40)
Professional attitude towards residents	4.67 (2.85–7.65)	2.36 (1.42–3.90)	13.81 (7.88–24.20)
Communication of goals	2.98 (1.89–4.68)	1.10 (0.74–1.65)	1.40 (0.96–2.04)
Evaluation of residents	1.61 (0.97–2.67)	2.76 (1.67–4.55)	0.72 (0.45–1.17)
Feedback	2.45 (1.43–4.22)	1.11 (0.68–1.81)	2.20 (1.28–3.19)
*Surgery* *			
Learning climate	5.06 (1.56–16.44)	2.22 (0.90–5.50)	1.00 (0.39–2.59)
Professional attitude towards residents	4.08 (1.54–10.82)	1.83 (0.71–4.69)	9.60 (3.80–24.29)
Communication of goals	2.39 (0.97–5.90)	1.81 (0.82–4.03)	0.79 (0.35–1.76)
Evaluation of residents	1.14 (0.39–3.36)	2.63 (1.17–5.94)	2.31 (0.75–7.08)
Feedback	7.74 (2.52–23.79)	2.37 (0.87–6.48)	9.49 (3.09–29.08)

All models are adjusted for residents' residency training year and sex and for faculty's experience and sex.

* = adjusted for hospital.

# = adjusted for specialty.

In general the odd ratio represents the chance that a faculty improves by one point on the outcome variable (being seen as a role model), if he/she improves by one point on the predictor variable (teaching performance). For example, the odd ratio for the adjusted association between *overall teaching performance* and the role of physician for anesthesiology faculty is 9.7. This signifies that the chance is 9.7∶1 that this faculty is being regarded as a better physician role model by the residents, if this faculty improves his *overall teaching performance* by one point. Further we point out that these odd ratios, like odd ratios in general, have a logistic scale. Consequently, the ostensible wide variation in odd ratios in table 3, represent a considerably lower amount of variation when one should recalculate them into chance percentages.

## Discussion

### Main Findings

In the continuous search for better understanding and improving role modelling as a teaching strategy in residency training, a clear perspective is needed on the determinants of clinical faculty being perceived by residents as a role models. This study was set out to explore if faculty's role modelling is influenced by their teaching performance and if this occurs similarly across specialties. The results of this study present empirical evidence of the great influence of faculty's *overall teaching performance* on being seen as a role model teacher/supervisor, physician and person. Further, we found that the influence of the specific domains of teaching on the role modelling typologies varied widely across specialties. Overall, *professional attitude towards residents* and *feedback* were the strongest predictors of the teacher/supervisor and the person role, while *professional attitude towards residents* and *evaluation of residents* were the strongest predictors of the physician role.

### Strengths and Limitations of this Study

The multicenter approach that included both academic and non-academic teaching hospitals and the fact that close to 100% of the faculty of the educational teams included in this study were evaluated, imply that the study population represents a valid sample of Dutch clinical faculty. Further, the observed numbers of residents' evaluations completed per faculty were adequate for sufficient reliability of the SETQ evaluation data for anesthesiology, obstetrics and gynecology and surgery [11], [12]. For internal medicine and pediatrics, the numbers of evaluations completed per faculty, although, on average, lower than five, were close to the recently reported analysis that showed that 2 to 4 evaluations per faculty could still yield high reliability data (Cronbach's alpha >0.88) [10], [12]. We must also be cautious in transferring the results to other specialties or health care systems [16]. Note that for anesthesiology and internal medicine only faculty of one academic hospital were included in the study.

Further, the presumed causal relationship between teaching performance and role modelling was based on theory [1]. However, we cannot exclude the possibility that the associations found in this study are caused by a reverse relationship between teaching performance and role modelling or by a triangular relationship with a variable we did not measure in this study.

### Explanation of the Results

The results regarding faculty's *overall teaching performance* are in line with previous studies that suggest improved role modelling when faculty's teaching performance was enhanced [1], [3], [17].

Some domains of teaching performance simultaneously influenced residents' perception of all faculty's role model types, while others had a positive influence on just one specific role model type, while having hardly no influence on another role model type. An explanation may be the definitions of the domains of teaching performance: For example, the domain *professional attitude towards residents* includes approachability, listening attentively and being respectful toward residents, a broad definition [11]. Hence, it is perhaps not surprising that it is generally the most influential predictor across all three role modelling types. The description of *evaluation of residents* by contrast [11], includes performances that are clearly more prone to influence the role of teacher/supervisor or physician compared to the person role [5], [9]. The negative relationships between some domains of teaching and the role modelling typologies of physician and person should be interpreted with some caution, given the large confidence intervals that include both positive and negative relationships. If there would be a negative relationship however, it is possible that residents regard faculty who regularly evaluate residents or who focus on the learning aspects of the residency training as more demanding faculty. This may explain why residents at some specialties report a negative relationship between the domain *evaluation of residents* and the role model type of person.

The differences across specialties as found in this study may result from the fact that the core competences that have to be learned and the way training is organized differs. In surgical residency training for example, residents spend a considerable amount of time in individual training in the operation theater to acquire appropriate technical skills. Direct performance feedback to residents will play a prominent role in this setting. Feedback provided during an operation in the operation theatre may have more impact on residents, compared to feedback given in more quiet settings. This hypothesis supports the finding that surgical residents regard feedback as a stronger influencer of the teacher/supervisor and the person role compared to other specialties.

Beside the differences in residency training programs, previous studies suggest that residents across specialties cannot be considered a homogeneous group [18]–[20]. The kind of role models residents are looking for could vary as a result of differences in residency training programs or residents' personality, interests or career motivation.

### Implications for Clinical Education, Research and Policy

In general the findings of this study underline the importance of the specialty specific context and show that role modelling cannot be regarded as a universal process across specialties. To improve good role modelling, teaching faculty can modify their teaching performance towards the specific goals they have set for themselves and the specific context they teach in. The good news is that many domains of teaching performance evaluated in this study are cognitive in nature, so they can be learned or improved by faculty who want to improve their role modelling as a teaching strategy. A logical first step for faculty who want to improve their teaching performance is to get insight into their current teaching performance. Valid and useful systems that could inform self-assessment could be of great interest to those faculty [21]. Future research could explore if teaching faculty who want to improve their role modelling as a teaching strategy, can succeed by improving their teaching performance.

### Conclusions

This study might help teaching faculty in understanding their role modelling better. Based on the reported findings a noteworthy recommendation could be that faculty should consider investing in enhancing specific domains of teaching performance as these domains are proven to be most influential in being seen as a specific type of role model by residents. The reported cross-specialty variations stress the complex processes of role modelling and highlight the importance of the specialty-specific context.

## Supporting Information

Appendix S1 **Overview of the items and scales of the SETQ questionnaires.** The items shared the same subject “During my residency in [specialty], my attending faculty generally…”. # = this item was in the SETQ questionnaires for Internal Medicine and Gynecology & Obstetrics. ## = this item was in the SETQ questionnaires for Anesthesiology, Pediatrics and Surgery.
(DOC)

Box S1 **Some characteristics of the role model typologies** [**5]**, [9****] **.**
(JPG)

Box S2 **Preceding text of typical role model skills in the role model items of the questionnaires.**
(JPG)

## References

[1] Cruess SR, Cruess RL, Steinert Y (2008). Role modelling–making the most of a powerful teaching strategy.. BMJ.

[2] Paice E, Heard S, Moss F (2002). How important are role models in making good doctors?. BMJ.

[3] Wright SM, Kern DE, Kolodner K, Howard DM, Brancati FL (1998). Attributes of excellent attending-physician role models.. N Engl J Med.

[4] Rauen KC (1974). The clinical instructor as role model.. J Nurs Educ.

[5] Ullian JA, Bland CJ, Simpson DE (1994). An alternative approach to defining the role of the clinical teacher.. Acad Med.

[6] Wright SM, Carrese JA (2002). Excellence in role modelling: insight and perspectives from the pros.. CMAJ.

[7] Ambrozy DM, Irby DM, Bowen JL, Burack JH, Carline JD (1997). Role models' perceptions of themselves and their influence on students' specialty choices.. Acad Med.

[8] Lombarts KM, Heineman MJ, Arah OA (2010). Good clinical teachers likely to be specialist role models: results from a multicenter cross-sectional survey.. PLoS One.

[9] Boor K, Teunissen PW, Scherpbier AJ, van der Vleuten CP, van de Lande J (2008). Residents' perceptions of the ideal clinical teacher–a qualitative study.. Eur J Obstet Gynecol Reprod Biol.

[10] Arah OA, Hoekstra JBL, Bos AP, Lombarts K (2011). New Tools for Systematic Evaluation of Teaching Qualities of Medical Faculty: Results of an Ongoing Multi-Center Survey.. PLoS One.

[11] Lombarts KM, Bucx MJ, Arah OA (2009). Development of a system for the evaluation of the teaching qualities of anesthesiology faculty.. Anesthesiology.

[12] van der Leeuw R, Lombarts K, Heineman MJ, Arah O (2011). Systematic Evaluation of the Teaching Qualities of Obstetrics and Gynecology Faculty: Reliability and Validity of the SETQ Tools.. PLoS One.

[13] Litzelman DK, Westmoreland GR, Skeff KM, Stratos GA (1999). Factorial validation of an educational framework using residents' evaluations of clinician-educators.. Acad Med.

[14] Brasher AE, Chowdhry S, Hauge LS, Prinz RA (2005). Medical students' perceptions of resident teaching: have duty hours regulations had an impact?. Ann Surg.

[15] Arah OA, Heineman MJ, Lombarts K (2012). Factors Influencing Residents' Evaluations of Faculty Teaching Qualities and Role Model Status.. Med Educ.

[16] Zibrowski EM, Myers K, Norman G, Goldszmidt MA (2011). Relying on others' reliability: challenges in clinical teaching assessment.. Teach Learn Med.

[17] Maker VK, Curtis KD, Donnelly MB (2004). Are you a surgical role model?. Curr Surg.

[18] Buddeberg-Fischer B, Klaghofer R, Abel T, Buddeberg C (2006). Swiss residents' speciality choices–impact of gender, personality traits, career motivation and life goals.. BMC Health Serv Res.

[19] Hoffman BM, Coons MJ, Kuo PC (2010). Personality differences between surgery residents, nonsurgery residents, and medical students.. Surgery.

[20] Hojat M, Zuckerman M (2008). Personality and specialty interest in medical students.. Med Teach.

[21] Sargeant J, Armson H, Chesluk B, Dornan T, Eva K et al (2010). The processes and dimensions of informed self-assessment: a conceptual model.. Acad Med.

