# Supplementary material for: The Teacher, the Physician and the Person: How Faculty's Teaching Performance Influences Their Role Modelling
Source: PLoS One. 2012 Mar 12;7(3):e32089. doi: 10.1371/journal.pone.0032089 (PMC3299651; doi:10.1371/journal.pone.0032089)
Supplement: Appendix S1 — Overview of the items and scales of the SETQ questionnaires. The items shared the same subject “During my residency in [specialty], my attending faculty generally…”. # = this item was in the SETQ questionnaires for Internal Medicine and Gynecology & Obstetrics. ## = this item was in the SETQ questionnaires for Anesthesiology, Pediatrics and Surgery. (DOC) [file pone.0032089.s001.doc]

**Appendix S1**: Overview of the Items and Scales of the SETQ Instruments

| Item number | Scale  and items |
| --- | --- |
|  |  |
|  | *Learning climate* |
| L1 | Encourages residents to participate actively in discussions |
| L2 | Stimulates residents to bring up problems |
| L3 | Motivates residents to study further |
| L4 | Stimulates residents to keep up with the literature |
| L5 | Prepares well for teaching presentations and talks |
|  | *Professional attitude towards residents* |
| P1 | Listens attentively to residents |
| P2 | Is respectful towards residents |
| P3 | Is easily approachable during on-calls |
| P4^#^ | Is easily approachable for consultation |
| P5^##^ | Is easily approachable for consultation on the outpatients |
|  | *Communication of goals* |
| C1 | States learning goals clearly |
| C2 | States relevant goals |
| C3 | Offers to conduct a mini-CEX (clinical examination exercise) regularly |
| C4 | Repeats stated learning goals periodically |
|  | *Evaluation of residents’ knowledge and skills* |
| E1 | Evaluates residents’ specialty knowledge regularly |
| E2 | Evaluates residents’ analytical abilities regularly |
| E3 | Evaluates residents’ application of knowledge to specific patients regularly |
| E4 | Evaluates residents’ medical skills regularly |
|  | *Feedback* |
| F1 | Regularly gives positive feedback to residents |
| F2 | Gives corrective feedback to residents |
| F3 | Explains why residents are incorrect |
| F4 | Offers suggestions for improvement |

The items shared the same subject “During my residency in [specialty], my attending faculty generally…”

# = this item was in the SETQ instruments for Internal Medicine and Gynecology & Obstetrics

## = this item was in the SETQ instruments for Anesthesiology, Pediatrics and Surgery.
