# Supplementary figures and images for: The Teacher, the Physician and the Person: How Faculty's Teaching Performance Influences Their Role Modelling
Source: PLoS One. 2012 Mar 12;7(3):e32089. doi: 10.1371/journal.pone.0032089 (PMC3299651; doi:10.1371/journal.pone.0032089)

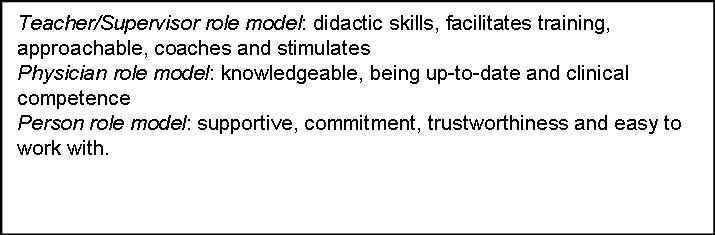

Supplement: Box S1 — Some characteristics of the role model typologies [5] , [9] . (JPG) [file pone.0032089.s002.jpg]

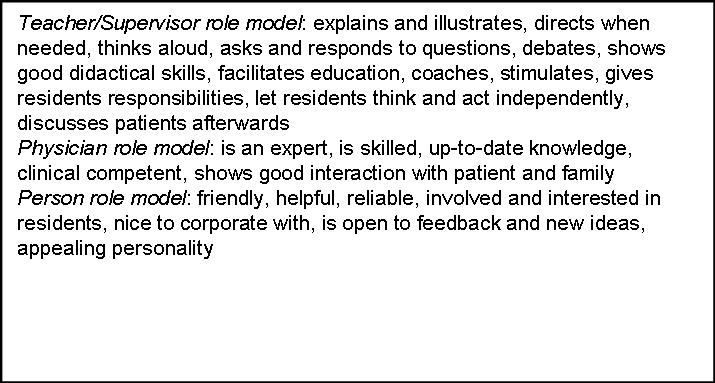

Supplement: Box S2 — Preceding text of typical role model skills in the role model items of the questionnaires. (JPG) [file pone.0032089.s003.jpg]
